# Osmolality and Tonicity of Isotonic Beverages

**DOI:** 10.3390/foods13101483

**Published:** 2024-05-10

**Authors:** Tjaša Skarlovnik, Andraž Lamut, Gregor Hostnik, Boris Gole, Urban Bren

**Affiliations:** 1 Faculty of Chemistry and Chemical Engineering, University of Maribor, Smetanova 17, SI-2000 Maribor, Slovenia; tjasa.skarlovnik@um.si (T.S.); gregor.hostnik@um.si (G.H.); 2 Meditop d.o.o., Ulica Vita Kraigherja 5, SI-2000 Maribor, Slovenia; andraz.lamut@meditop.si; 3 Institute for Environmental Protection and Sensors, d.o.o., Beloruska ulica 7, SI-2000 Maribor, Slovenia; 4 Faculty of Medicine, University of Maribor, Taborska ulica 8, SI-2000 Maribor, Slovenia; boris.gole@um.si; 5 Faculty of Mathematics, Natural Sciences and Information Technologies, University of Primorska, Glagoljaška 8, SI-6000 Koper, Slovenia

**Keywords:** hydration, osmolality, tonicity, isotonic beverages, erythrocytes

## Abstract

This study aimed to measure and compare the osmolality and tonicity of isotonic beverages that can be bought on the Slovenian market. The main goal was to examine how good is the agreement between the measured osmolalities of the beverages and the requirements for isotonic beverages set up by EFSA. Osmolalities were measured with an osmometer using the freezing point depression method. Afterwards, two complementary methods for the observation of tonicity were developed. Erythrocytes were exposed to standard NaCl solutions of different osmolalities to observe their influence on the volume and shape of cells following the turbidity of the solution and the morphology of erythrocytes. These two methods enabled us to determine whether standard solutions were hypo-, iso-, or hypertonic. In this way, we found that the osmolality of 12 out of the 18 investigated isotonic beverages was in the range of 270–330 mOsm/kg, as required by EFSA. However, six samples did not meet this criterion and should therefore not have the label “isotonic” or be described as such. The measurements of turbidity of solutions indicated that most isotonic beverages exhibit a lower tonicity than standard NaCl solutions of identical osmolality. However, examination of the erythrocytes in isotonic beverages showed that the measurements were additionally complicated by the low pH values of these beverages. Finally, by demonstrating how different components of isotonic beverages pass through the erythrocyte membranes, we found that even isoosmolal beverages are often not isotonic, as the concentration of actively transported sugars in these beverages is relatively high.

## 1. Introduction

Isotonic sports drinks are well-known among athletes and widely used to achieve proper hydration of the body and to increase training capacity because of their high electrolyte and carbohydrate content and subsequent ergogenic properties [1]. In the last 20 years, these drinks have gained popularity among athletes and the food industry, resulting in an overall global increase in the production and availability of such drinks on the market [2]. In general, sports drinks are classified into three different categories, depending on their osmolality and the amount of carbohydrates and electrolytes used [3,4]. (I) Hypotonic sports drinks usually contain lower amounts of carbohydrates (<3 g/100 mL) and have osmolality below 270 mOsm/kg. They are used in conditions where rapid rehydration is required with the specific aim to replenish the lost fluids and when the athlete’s demand for carbohydrates during exercise is low (e.g., during short-term exercises). (II) Isotonic drinks contain carbohydrates in the range of 6–8 g/100 mL and have osmolality between 270 and 330 mOsm/kg, which corresponds to the osmolality of bodily fluids. (III) Hypertonic sports drinks have osmolality above 330 mOsm/kg and include an even higher percentage of carbohydrates, which further contributes to the caloric content of these drinks and slowing down the absorption of water in the intestine. Therefore, hypertonic drinks are mainly used for carbohydrate loading in the case of strenuous and prolonged exercises. Sports drinks usually contain around 20–30 mmol/L of sodium as the basic electrolyte that enables sufficient rehydration and replenishment of sodium lost in the sweat. On the other hand, consumed carbohydrates provide fuel for the working muscles when liver and muscle glycogen storage is depleted, such as during prolonged exercise [1,5]. As declared by the European Food Safety Authority (EFSA), isotonic beverages must have an osmolality between 270 and 330 mOsm/kg to be designated “isotonic”, which in this context refers to the osmolality of bodily fluids [6]. It is, therefore, not surprising that the word isotonicity is often used somehow interchangeably with isoosmolality in the field of sports science [7,8]. However, osmolality and tonicity represent two different parameters from the physicochemical point of view, which are closely related to the osmotic pressure of cells in the physiological environment. To understand the difference between these two quantities, we must, therefore, first provide the definition of osmotic pressure.

Osmotic pressure is a consequence of the spontaneous transport of solvent through a semipermeable membrane due to the presence of non-penetrating solute in the solution on one side of this membrane. The equilibrium is reached when the hydrostatic pressure on the other side of the membrane is the same as the osmotic pressure, which is the driving force pushing the solvent across the semipermeable membrane [9]. The osmotic pressure (Π) can be calculated using Equation (1):

(1) Π=R⋅T⋅m,

where *R* is the ideal gas constant, *T* is the temperature, and *m* is the osmolality of the solution, defined as the product of the molality of the solution (*b*) and the number of particles that a given compound dissociates into the solution (ν), given by Equation (2):

(2) m=b⋅ν,

Finally, not all of the solutions that one encounters are ideal. The osmotic coefficient (Φ) gives the deviation of the non-ideal solution’s colligative properties (i.e., properties that depend only on the molar concentration of solutes and not on their types) from the ideal solution’s colligative properties. After combining all of these factors, Equation (3) for osmotic pressure reads:

(3) Π=R⋅T⋅b⋅ν⋅Φ,

Tonicity (“effective osmolality” [10]) is a quantity partly related to osmolality. While osmolality represents purely a physicochemical quantity, tonicity is more physiological, which strongly depends on the properties of the physiological membrane of interest. Although the difference between the two quantities is well-known [11], they are still often erroneously used interchangeably.

Let us consider two solutions, one being the aqueous NaCl solution and the other the aqueous urea solution, to demonstrate the difference between the two quantities. Both solutions are isoosmolal—e.g., the osmolality of both solutions is 300 mOsm/kg. The cell shape indeed remains intact when erythrocytes are placed into the first solution. This means the first isoosmolal solution is also isotonic due to a solute that cannot penetrate the erythrocyte membrane. On the contrary, urea can penetrate the erythrocyte membrane, effectively increasing the osmolality of the erythrocyte’s interior [12]. Consequently, the solvent is also transferred into erythrocytes, which leads to an increase in the erythrocyte cell volume and eventually to the lysis of erythrocytes due to the high osmotic pressure. Therefore, one can conclude that the second solution (isoosmolal aqueous solution of urea) is not isotonic due to the penetrating nature of the solute. The same holds true for glucose solutions [12,13].

The present work investigated the representative samples of “isotonic beverages” on the Slovenian market. Initially, the osmolality of isotonic beverages was measured, followed by developing a method for determining the tonicity of samples. Finally, the application of the developed method on examined isotonic drinks was demonstrated. At the same time, it was shown how certain components of isotonic beverages can penetrate erythrocyte cell membranes and, therefore, do not contribute to the tonicity of beverages.

## 2. Materials and Methods

In this study, 18 samples, representing the largest market share of sports drinks with the label “isotonic” available in the Slovenian market, were analyzed. Samples were obtained from the most popular local supermarkets in January 2022 and selected according to their popularity and availability for the final consumer.

### 2.1. Osmolality

The osmolality of isotonic beverages was measured using the freezing point depression with an osmometer (Knauer, Berlin, Germany) and the recorder Flatbed (Kipp & Zonen, Delft, The Netherlands). Calibration of the instrument was performed using distilled water and a standard sodium chloride solution with an osmolality of 400 mOsm/kg (Knauer, Berlin, Germany) before the analyses. Isotonic beverages were shaken and pipetted (0.15 mL) into the test tube. Each measurement was repeated three times, and the average osmolality was calculated.

### 2.2. Isolation of Erythrocytes

The collection of human blood samples was approved by the National Medical Ethics Committee of the Republic of Slovenia (approvals nr. 0120-194/2019/4, issued 23 May 2019, 0120-187/2019/10, issued 27 June 2019 and 0120-602/2019/3, issued 6 February 2020). Written informed consent was obtained from all donors who participated in the study.

In total, 6 mL of venous blood was collected into K2E (EDTA) vacutainers (BD, Franklin Lakes, NJ, USA). Erythrocytes were then isolated through gradient centrifugation (1200× *g*, 10 min, RT) using density medium (1.094 g/mL, mixed from High Density Spin Medium and Density Diluent Medium, both pluriSelect, Leipzig, Germany) and SepMate50 tubes (Stem Cell Technologies, Vancouver, BC, Canada). Erythrocytes settled to the bottoms of the tubes, while plasma, platelets, and the white blood cells remained above the gradient medium. Erythrocytes were transferred into new tubes and centrifugated for 20 min at 300× *g* to remove any remaining platelets. Finally, clean erythrocytes were diluted 1:1 with Dulbecco’s Phosphate Buffered Saline (Sigma-Aldrich, Burlington, MA, USA).

### 2.3. UV/Visible Spectroscopy

All experiments were performed on a Cary50 UV/Vis spectrophotometer (Varian Inc., Mulgrave, Australia) equipped with a Single Cell Peltier Accessory (Varian Inc., Mulgrave, Australia). Experiments were performed in a cuvette with an optical path of 1 cm. All spectra were measured in a wavelength range between 800 and 200 nm, with 1 nm step and an averaging time of 0.2 s. The kinetics of erythrocyte lysis were observed at a wavelength of 780 nm, where the −log(*I*/*I*_0_) value was measured every 6 s.

The standard stock erythrocyte suspensions were prepared such that 0.1 mL of standard stock suspension added to the 3 mL of standard solution with an osmolality of 300 mOsm/kg resulted in a suspension with a −log(*I*/*I*_0_) value of 0.8 at a wavelength of 780 nm. Finally, 0.1 mL of this standard stock solution was added to the 3 mL of standard NaCl solution. Measurements were performed 20 min after the erythrocytes were exposed to the solutions.

Standard NaCl solutions with osmolalities of 100, 150, 200, 250, 275, 300, 325, 350, 400, 500, and 600 mOsm/kg were prepared, and UV/Vis spectra of erythrocyte suspensions in these solutions were measured to construct a calibration curve.

The kinetics of standard NaCl solutions were also measured according to the protocol described above at a wavelength of 780 nm. From the moment the erythrocytes were added to the standard solution to the start of the measurement, an average of 20 s passed, which allowed us to observe the speed of lysis in hypotonic solutions.

The UV/Vis spectra of isotonic drinks were measured to obtain information on how the absorption of light in the beverages can interfere with the tonicity determination. Afterwards, 0.1 mL of diluted erythrocytes was added to 3 mL of isotonic beverage previously filtered with an injection filter (PVDF; Millex-GV, Carrigtwohill, Ireland) with a pore size of 0.22 μm. After 20 min, the spectrum of erythrocyte suspension in isotonic beverages was measured.

The passage of saccharides through the cell membrane was examined using UV/Vis spectroscopy, as well. Standard solutions of D-glucose, D-fructose, and sucrose with the following osmolalities were prepared: 300, 325, 350, 400, 500 and 600 mOsm/kg. The measurements were carried out according to the same protocol as that used for the standard sodium chloride solutions at a wavelength of 780 nm.

### 2.4. Microscopy

Erythrocytes were observed with Cytation 5 Cell Imaging Multi-Mode Reader (Agilent Technologies, Inc., Santa Clara, CA, USA) under 20-times magnification. Erythrocytes were diluted in a ratio of 1:200 using the standard NaCl solution with an osmolality of 300 mOsm/kg. Then, 90 μL of standard NaCl solution was added to the 10 μL of diluted erythrocytes in a 96-well flat-bottom microplate (Corning Inc., Somerville, MA, USA). Pictures were taken 20 and 60 min after the erythrocytes were exposed to the solutions.

### 2.5. pH Measurements

pH values were measured using pH meter 780 (Metrohm, Herisau, Switzerland) with added magnetic stirrer 801. The pH meter/glass electrode was calibrated at two points using buffer solutions with pH 4 and 7. The calibrated electrode was then applied to measure the pH of isotonic drinks.

### 2.6. Atomic Absorption and Emission Spectroscopy

The content of Na^+^, K^+^, and Mg^2+^ ions was determined using an atomic optical spectrometer AOS (Perkin Elmer, Waltham, MA, USA). Standard magnesium solutions with a concentration of γ = 1000 mg/L and γ = 10 mg/L were prepared from an appropriate weight of magnesium nitrate. Then, we also prepared standard solutions with concentrations of 0.5, 1.0, 1.5, 2.0, and 2.5 mg/L, and we measured their absorption *A* at a wavelength of 285.2 nm and an analyte flow rate of 5 mL/min and then constructed a calibration curve. The measurements of Mg^2+^ ions in isotonic beverages followed.

First, we prepared a standard NaCl solution with a concentration of γ = 1000 mg/L, from which a further solution with a concentration γ = 10 mg/L was diluted. Standards for the calibration curve were prepared from this solution with sodium ion (Na^+^) concentrations of 0.2, 0.4, 0.6, 0.8, and 1.0 mg/L. Afterwards, the emission intensity (*I*_E_) of the standard solutions at 589 nm was recorded, and a calibration curve was constructed. Subsequently, the concentration of sodium ions in the isotonic beverages was measured. An analogous procedure was applied to measure potassium ions (K+) at a wavelength of 766.5 nm, and their concentrations in isotonic drinks were obtained based on a calibration curve constructed from standard KCl solutions.

## 3. Results

### 3.1. Osmolality

The osmometer was calibrated at two points (0 mOsm/kg and 400 mOsm/kg), and the measured osmolalities of isotonic beverages are reported in Table 1. The experimental error was estimated using Student’s *t*-test for three measurements. The osmolalities of all 18 samples lay within the calibration range. Twelve samples (1, 2, 3, 4, 5, 7, 8, 10, 11, 15, 16, and 17) meet the required regulative range of osmolality, as declared by EFSA (270–330 mOsm/kg). Samples 9 and 18 are slightly hypoosmolal, 6 and 13 are hypoosmolal, and 12 is strongly hypoosmolal. Sample 14 is slightly hyperosmolal (342 mOsm/kg).

### 3.2. Determination of Tonicity through UV/Visible Spectroscopy

**Figure 1:**
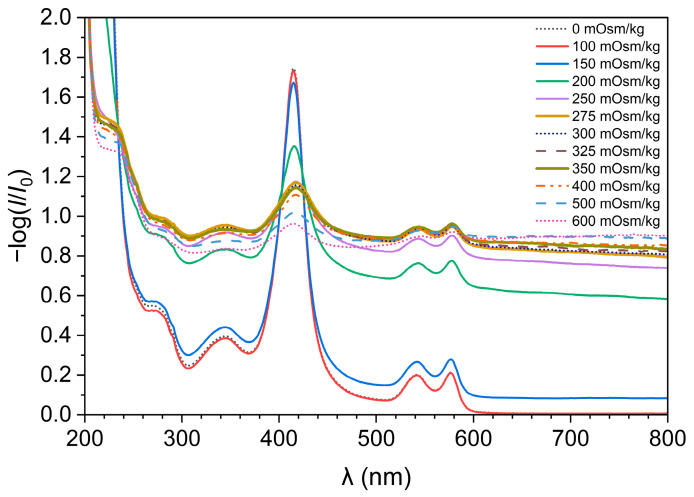
Spectra of erythrocyte suspension in standard NaCl solutions of different osmolalities.

We examined how the solutions with different osmolality affect the cell structure by exposing the erythrocytes to 12 standard NaCl solutions of different osmolalities (0, 100, 150, 200, 250, 275, 300, 325, 350, 400, 500, and 600 mOsm/kg—Figure 1). The UV/Vis spectra were measured 20 min after exposing erythrocytes to the standard solutions. The graph (Figure 1) depicts the decadic logarithm of the ratio between the light that passed through the blank sample (water) *I*_0_ and the light that passed to the detector through the solutions of erythrocytes *I* as a function of wavelength. The decrease in the intensity of the light that reaches the detector when erythrocytes are present is the effect of light absorption and scattering by the cells, while after the lysis of erythrocytes, the obtained spectrum is a consequence of light absorption by hemoglobin.

On the spectra (Figure 1), we can see five distinctive peaks at the wavelengths of 270, 345, 416, 543, and 577 nm. These peaks result from the presence of oxygenated, free hemoglobin [14,15]. The peaks are less pronounced in erythrocyte suspensions of high osmolality, which is most noticeable at the wavelength of 416 nm. This is a consequence of the fact that in the high osmolality solution, the hemoglobin is packed in the erythrocytes. Because no other compound in the solution absorbs light at wavelengths between 700 nm and 800 nm, the −log(*I*/*I*_0_) values result from the light scattering by erythrocytes. Solutions with an osmolality of 0 mOsm/kg and 100 mOsm/kg have a value of −log(*I*/*I*_0_) 0 at a wavelength of 780 nm, which means that the erythrocytes in both solutions were completely lysed (Figure S1). As a result, we can also see more pronounced absorption peaks from lysed cells in the spectra because of the released hemoglobin.

**Figure 2:**
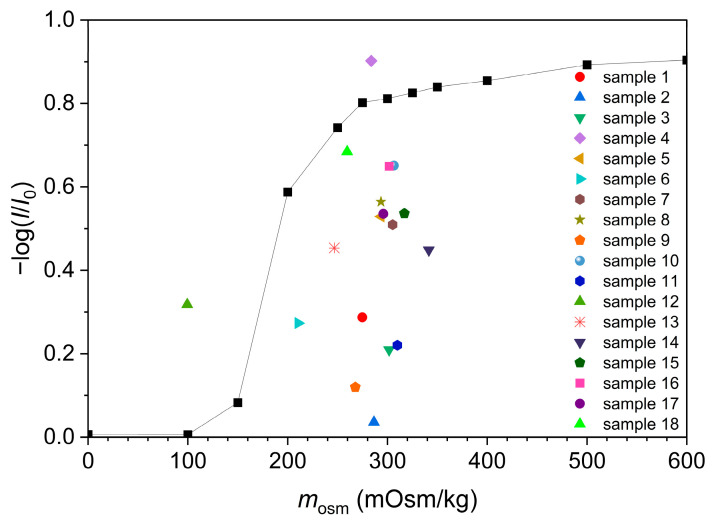
Calibration curve −log(*I*/*I*_0_) as a function of osmolality (black square symbols) with added values of isotonic drinks.

A calibration curve in Figure 2 was drawn from the measured spectra of erythrocyte suspensions in standard NaCl solutions at a wavelength 780 nm and at the time of 20 min, and it shows the value of the −log(*I*/*I*_0_) as a function of the solution’s osmolality. Therefore, we can assume that the tonicity of standard NaCl solutions is the same as their osmolality. In Figure 2 and Figure S1, we can see that a NaCl solution with an osmolality of 275 mOsm/kg is already detected as isotonic because the differences in the −log(*I*/*I*_0_) values of solutions with osmolality equal to or greater than 275 mOsm/kg are small.

Moreover, on the calibration curve (Figure 2) were added values of −log(*I*/*I*_0_) of erythrocytes in isotonic beverages as a function of the osmolality of these beverages at a wavelength of 780 nm. For most of the isotonic beverages, it appears that their tonicity is lower than the tonicity of standard NaCl solutions of identical osmolality (Figure 2), which is likely a consequence of the presence of penetrating solutes.

**Figure 3:**
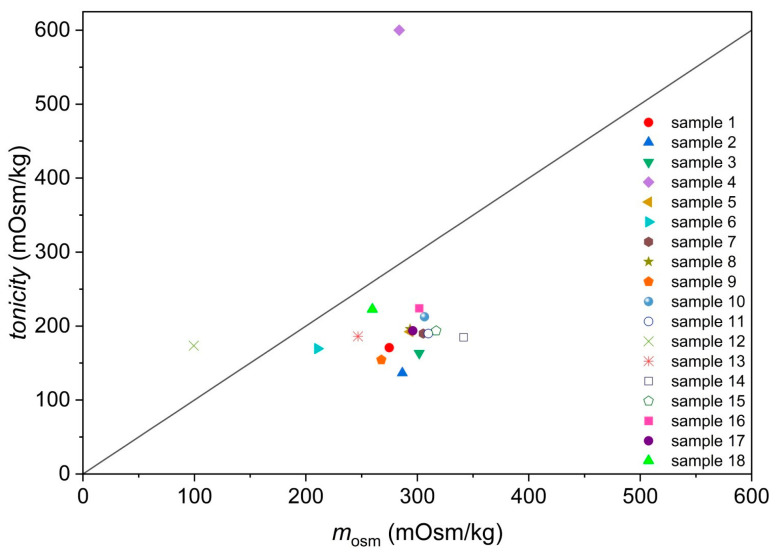
Osmolality versus apparent tonicity of isotonic drinks as determined by observing lysis of erythrocytes with UV/Vis spectroscopy.

Similar information can be obtained from Figure 3, where the comparison between the measured osmolalities and apparent tonicities of isotonic beverages is depicted. Apparent tonicities of isotonic beverages are determined as the osmolality of the aqueous NaCl solution possessing an equal −log(*I*/*I*_0_) value to the isotonic beverage. In two samples (samples 4 and 12), the tonicity determined through UV/Vis spectroscopy seems larger than their osmolalities. This might be a consequence of a changed morphology of erythrocytes and cell membrane permeability in low-pH solutions [16,17,18].

**Figure 4:**
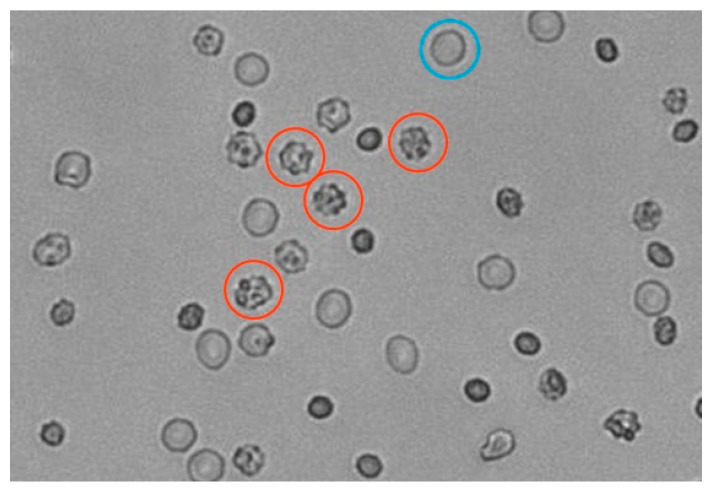
A normal cell (blue) and a crenation cell (red).

**Figure 5:**
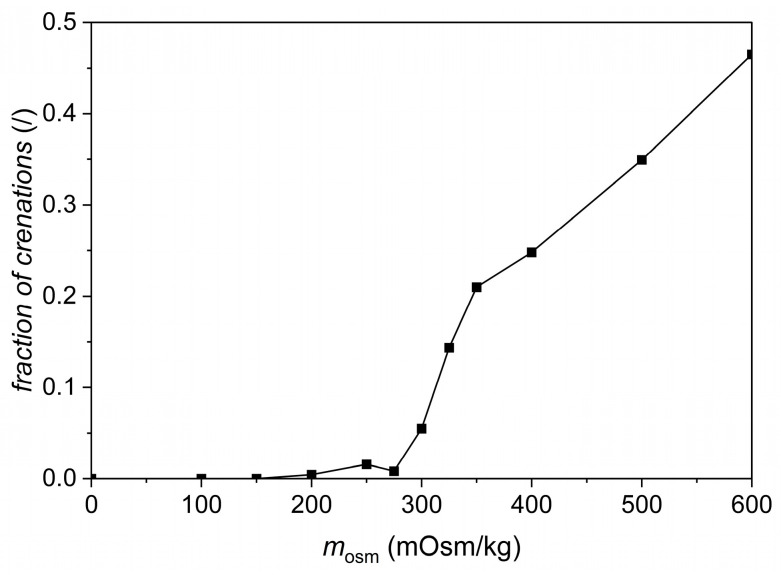
The fraction of crenations in standard NaCl solutions of different osmolalities.

Unfortunately, the developed method is not sensitive enough for hypertonic solutions. To find the cause of the discrepancy between osmolality and tonicity in beverages and to obtain a tool to distinguish between isotonic and hypertonic solutions, we therefore applied a multimodal imaging reader to observe the shape of erythrocytes in various solutions (Figure 4). The observation of cells was performed 20 min after the addition of erythrocytes into the solutions (Figures S4–S15). All measurements were performed in triplicate for better reproducibility, as fluctuations in the number and morphology of erythrocytes in the picture may occur in individual samples due to the exact location of sampling (image capture). Initially, the calibration curve, depicted in Figure 5, was constructed. In Figure 5, the fraction of the cells that were morphologically changed into crenations (Figure 4) is depicted as a function of the osmolality of standard NaCl solutions. One can see that the crenations were not present in the NaCl solutions with osmolalities up to 200 mOsm/kg. Contrary to expectations, a small fraction of crenations can also be observed in NaCl solutions with osmolality between 200 and 300 mOsm/kg. This fraction increases steeply with increasing osmolality in NaCl solutions with osmolalities between 300 mOsm/kg and 350 mOsm/kg and more gradually at even higher osmolalities. This enables us to also discriminate between isotonic and hypertonic solutions due to the increasing fraction of crenations with increasing osmolality/tonicity.

**Figure 6:**
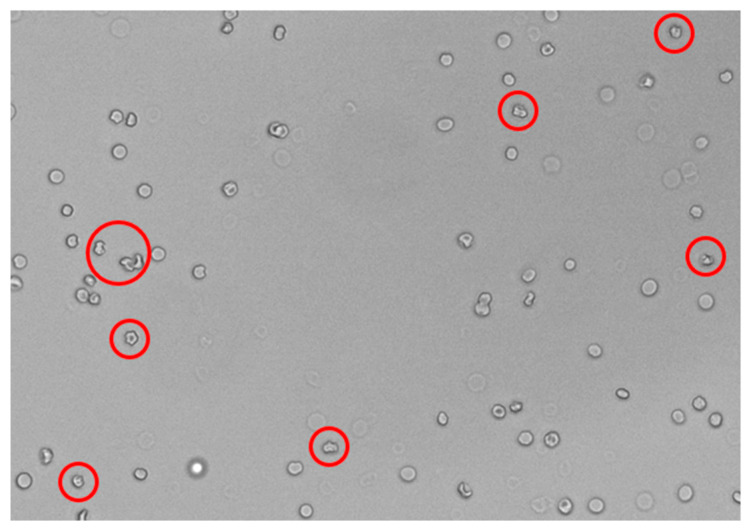
Deformed cells (in red circles) in isotonic beverage sample number 1.

We tried to apply the same method to the isotonic beverages. Figure 6 shows erythrocytes in a sample of isotonic 1 with osmolality of 275 mOsm/kg and, according to the tonicity measurements (Figure 3), this sample belongs to the hypotonic range. Still, a lot of deformed cells can be seen. This is most likely a consequence of low pH values of isotonic beverages that affect the erythrocytes’ morphology [16,17].

**Figure 7:**
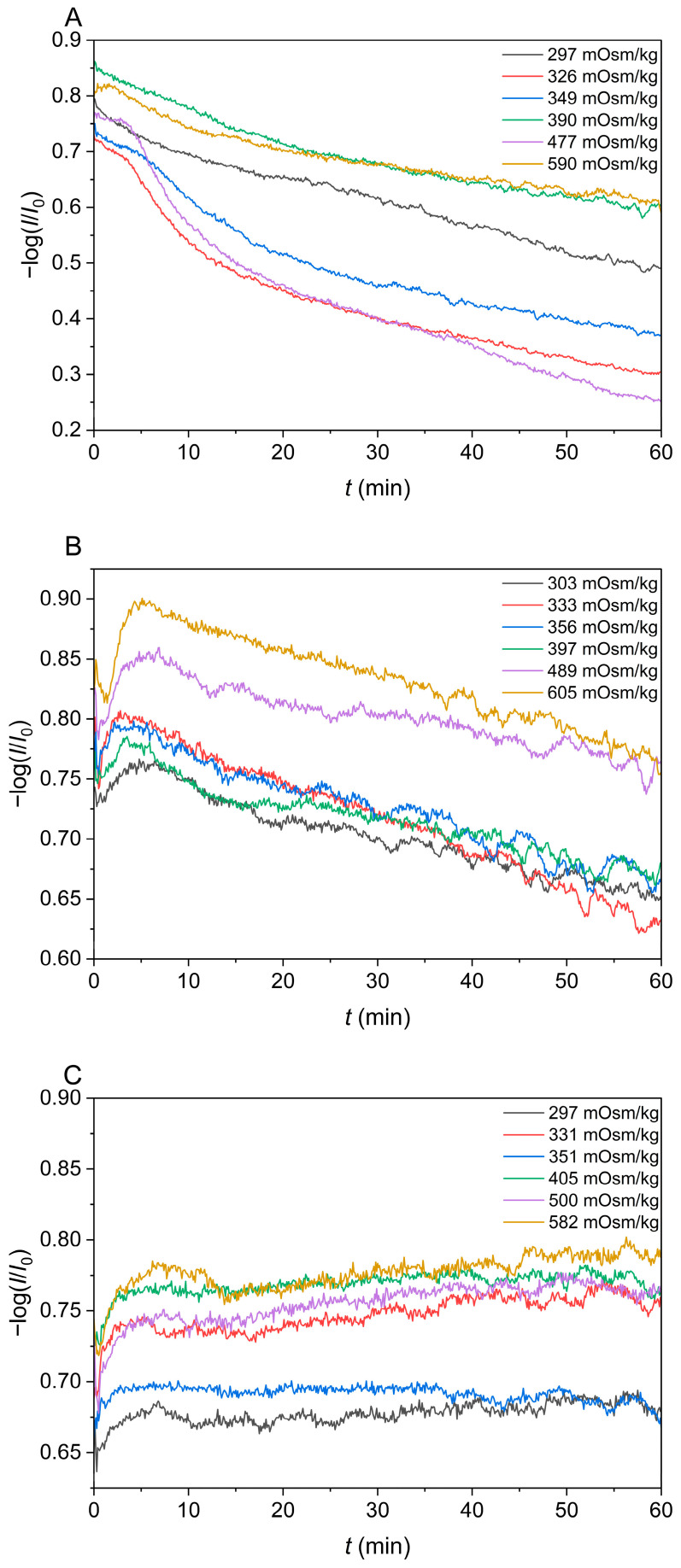
Kinetics of transport of (**A**) glucose, (**B**) fructose, and (**C**) sucrose across the erythrocyte membrane at a wavelength 780 nm.

According to the literature data, certain ingredients in isotonic beverages are able to pass through the erythrocyte membrane. Therefore, we decided to demonstrate how the carbohydrates, which are found as common ingredients in isotonic beverages, pass through the cell membrane. The time dependence of −log(*I*/*I*_0_) was measured to examine this effect, and the corresponding results are depicted in Figure 7. One can see that −log(*I*/*I*_0_) relatively quickly decreases with time in the case of glucose (Figure 7A), while this response is less pronounced but still present in the case of fructose (Figure 7B). On the contrary, the value of −log(*I*/*I*_0_) remains constant in the case of sucrose, meaning that sucrose is unable to effectively pass the erythrocyte membrane.

Because glucose and fructose cross the cell membrane [12,13,19,20], they contribute to the osmolality of the solution but not to its tonicity. This means that all standard glucose solutions were hypotonic, which is confirmed by decreasing values of −log(*I*/*I*_0_) in Figure 7A. However, one can see that the lysis of cells (decrease in −log(*I*/*I*_0_) value) is slower than the lysis in a hypoosmolal NaCl solution, because the factor that determines the speed of cell lysis here is the rate of solute (glucose) passage through the cell membrane and not only the rate of water transfer through the erythrocyte membrane. Comparing graphs in Figure 7A,B, we can see that the diffusion of fructose through the cell membrane is slower than the diffusion of glucose. On the contrary, sucrose is not able to pass the erythrocyte membrane (Figure 7C).

Table 2 reports the average fractions of normal and crenation cells after 20 and 60 min in glucose, fructose, and sucrose solutions with an osmolality of approximately 300 and 500 mOsm/kg. This confirms the results from the UV/Vis spectroscopy and indicates that the fraction of crenations for hyperosmolal glucose and fructose solutions is not as high as in NaCl solutions (Figure 5). If we examine sucrose 500 mOsm/kg solutions, the fraction of crenations is nearly the same as in the corresponding NaCl solutions. On the contrary, one can see that the fraction of crenations is significantly smaller in glucose and fructose solutions of osmolality around 500 mOsmol/kg, which is a consequence of the ability of these solutes to pass the erythrocyte membranes.

## 4. Discussion

Isotonic beverages are used in sports with the aim of providing proper hydration to the body. Because the food industry is not as regulated as, e.g., the pharmaceutical industry, the first goal of the present study was to examine if the osmolalities of isotonic beverages indeed comply with the EFSA regulations. These require that the osmolality of isotonic beverages is between 270 and 330 mOsm/kg [6,21]. In total, 12 investigated samples met this criterion, but 6 investigated samples did not meet the requirements, and they should, therefore, not be labelled as isotonic. The most significant deviation was observed in sample 12, whose osmolality was only 99 mOsm/kg. This is somehow not surprising given the nutritional specification of the sample, as, according to the declaration, it contains neither carbohydrates nor sugars, which usually largely contribute to the osmolality of isotonic beverages (Table S1). It also contains negligible amount of salt according to the declaration, which means that it contains a very low quantity of substances that actually contribute to the osmotic pressure. However, our experimental data (Table S2) show a much higher value of the Na^+^ ions (88 mg/100 mL or 38.4 mmol/L). If we assume that Na^+^ ions are added as a simple 1:1 salt (e.g., sodium chloride), the estimated salt contribution to osmolality would be approximately 77 mOsm/kg, which represents a reasonable explanation for the measured osmolality.

Moreover, we wanted to determine how tonicities of samples correspond to their osmolalities. This was performed through the observation of erythrocytes’ behavior in various solutions. Erythrocytes were used for measurements and the determination of tonicity because they are easily available and they can be applied to suspensions, contrary to other cells. Initially, the calibration curves using standard NaCl solutions of different osmolalities were constructed, which enabled us to estimate whether the solution was hypotonic (UV/Visible spectroscopy) or hypertonic (microscopy). Through the combination of both methods, it is possible to estimate the tonicity of a given solution. UV/Visible spectroscopy is useful to assess if the solution is hypotonic, and it is easier to process the results and the statistical capture of the solution’s properties. On the other hand, we cannot firmly estimate the limit between isotonic and hypertonic solutions. Meanwhile, the rough limit between isotonic and hypertonic solutions can be obtained by using microscopy with the multimodal reader, where we can also observe changes in cell shapes. However, it is not possible to observe and estimate hypotonicity with microscopy because the number of lysed cells cannot be quantified. Due to the influence of sampling (image capture), the statistical analysis is inferior to UV/Vis spectroscopy, as we observe a smaller number of cells.

Isotonic beverages are often colored solutions with many dye molecules and other ingredients, which might interfere with the measured erythrocyte light scattering. The spectra of filtered solutions were examined (as depicted in Figure S2), and it was observed that none of the isotonic beverages absorbed the light at wavelengths between 700 and 800 nm, although the isotonic beverages 5, 11, and 17 absorb light up to a wavelength of 700 nm (Figure S2). Consequently, for the determination of tonicity, any wavelength in the range between 700 nm and 800 nm could be chosen. This wavelength range thus facilitated the determination of tonicity. Therefore, the wavelength of 780 nm was chosen because 780 nm is far enough away from the absorption of each component of the solution. Apparently, the tonicities of almost all of the investigated samples (with the exception of samples 4 and 12) were lower than their corresponding osmolalities. However, the composition of the samples is rather complex, and various components of isotonic beverages might affect the morphology of the erythrocytes. Moreover, the pH values of all isotonic beverages are acidic (see Table S3), and the pH value indeed affects the morphology of erythrocytes [16,17], resulting in the invagination of erythrocytes (see Figure 6). This might be the reason behind the high values of estimated tonicity for samples 4 and 12 [16]. The low pH might have also influenced the remaining measured osmolalities. One possible solution for a given problem would be the adjustment of the pH of isotonic beverages before measurement. However, this adjustment is not possible without significantly changing the osmolality of the isotonic beverages.

To sum up, the main difference between osmolality and tonicity is that all non-volatile solutes in the solution contribute to osmolality, such as, for instance, carbohydrates, while only solutes that do not cross the cell membrane, such as, e.g., inorganic ions (Na^+^, K^+^, Cl^−^, Ca^2+^, and Mg^2+^) contribute to tonicity [12,13,19,22,23,24,25]. Consequently, we tried to find out how individual components of isotonic beverages pass the cell membrane and contribute to tonicity. Salts contribute to both osmolality and tonicity [13,22,23,24,25], while the remaining important component of isotonic beverages, carbohydrates, can, to a certain extent, pass through physiological membranes. That is why we decided to check how sugars, which are often found in isotonic beverages, pass through the erythrocyte membrane. The transport of sugars across the cell membrane depends on the permeability of the membrane or its ability to transport them. Glucose transport through the erythrocyte membrane is regulated by glucose transporters GLUT-1 (glucose transporter protein 1), [26] which can be seen in our experiments by a decreasing value −log(*I*/*I*_0_) as a function of time (Figure 7A). The transport of fructose across the cell membrane is regulated by transporter GLUT-5 [13,26]. The results of UV/Vis spectroscopy (Figure 7A,B) indeed confirmed that standard glucose and fructose solutions are hypotonic. Meanwhile, a stable value of −log(*I*/*I*_0_) (Figure 7C) and a relatively high fraction of crenations in a 500 mOsm/kg sucrose solution (which is close to their fraction in standard NaCl solutions) prove that sucrose does not cross the erythrocyte membrane (see Table 2 and Figure 5). In addition, a fraction of the crenations increases with the increasing osmolality of standard sucrose solutions, which could also be seen for standard NaCl solutions (Figure 5).

Many of the examined isotonic beverages contain various types of sugars, which is the basis of their ergogenic properties during sports activity. These sugars also largely contribute to the osmolality of isotonic beverages. However, as demonstrated in this study, simple monosaccharides, such as glucose and fructose, which are often found in these beverages, can pass the membranes of erythrocytes and, therefore, they do not contribute to the tonicity. Therefore, even if such beverages comply with the osmolality demanded by EFSA, such drinks could not be considered isotonic in the true meaning of this physiological parameter. Unfortunately, the labelled contents of examined beverages are often not detailed enough to estimate the contribution of these sugars to the osmolality and tonicity of samples (Table S1). Moreover, from Table S2, it can be seen that concentrations of electrolytes and, consequently, their contribution to the osmolality (especially samples 1, 2, 3, 11, 14, and 16) are relatively low, and therefore they contain high amounts of carbohydrates and sugars to reach isoosmolality (Table S1). Given that most samples contain glucose and/or fructose (Table S1), it is not surprising that their tonicity is lower than their osmolality. Finally, if the low pH value of isotonic beverages makes it difficult to determine the tonicity of beverages directly, the contribution to the tonicity of each individual component could be determined through the combination of methods used in this research, and the correlation between osmolality and tonicity can be established.

## 5. Conclusions

In the present study, we found that 12 out of 18 investigated isotonic beverages met the osmolality requirements set by EFSA for isotonic beverages. However, six investigated samples did not meet this criterion, which could be attributed to the poor regulatory requirements before the entry of isotonic beverages on the market. Most beverages exhibited lower apparent tonicity than standard NaCl solutions of comparable osmolalities. However, the determination of tonicity was somewhat affected by the low pH of isotonic beverages. Furthermore, we were able to demonstrate that two sugars, glucose and fructose, are able to pass the erythrocyte membrane. Thus, lower apparent tonicities are likely the result of beverages’ composition, as they usually contain a lot of sugars, which can pass through cell membranes and consequently do not contribute to the tonicity of beverages.

In conclusion, we experimentally demonstrated that tonicity and osmolality are two different quantities, which are often erroneously used interchangeably, and that glucose and fructose contribute to the osmolality, but not to the tonicity, of isotonic beverages. This information could be used in the further development of isotonic beverages. Moreover, the presence of samples that do not conform to EFSA regulations shows a need for better control of the isotonic beverages market in the future.

## Figures and Tables

**Table 1:** Osmolality measurements of isotonic beverages.

Sample	Average Osmolality (mOsm/kg)
1	275 ± 5.0
2	287 ± 5.0
3	302 ± 5.0
4	284 ± 7.0
5	294 ± 22
6	211 ± 17
7	305 ± 5.0
8	294 ± 13
9	268 ± 5.0
10	306 ± 10
11	310 ± 5.0
12	99 ± 5.0
13	247 ± 5.0
14	342 ± 5.0
15	317 ± 5.0
16	302 ± 5.0
17	296 ± 22
18	260 ± 5.0

**Table 2:** Fractions of normal cells and crenations in sugar solutions of different osmolalities.

Osmolality (mOsm/kg)/Time (min)	Type of Cells	20 min	60 min
297 mOsm/kg solution of glucose	normal cells	0.92	0.92
	crenations	0.08	0.08
477 mOsm/kg solution of glucose	normal cells	0.89	0.9
	crenations	0.11	0.1
303 mOsm/kg solution of fructose	normal cells	0.9	0.86
	crenations	0.1	0.14
490 mOsm/kg solution of fructose	normal cells	0.98	0.99
	crenations	0.01	0.01
297 mOsm/kg solution of sucrose	normal cells	0.82	0.77
	crenations	0.18	0.23
500 mOsm/kg solution of sucrose	normal cells	0.73	0.7
	crenations	0.27	0.3

## Data Availability

The data presented in this study are available on request from the corresponding author (accurately indicate status).

## Supplementary Materials

The following supporting information can be downloaded at: https://www.mdpi.com/article/10.3390/foods13101483/s1, Table S1: Labelled content of nutrients per 100 mL of isotonic beverage; Table S2: Experimentally measured Na^+^, K^+^, and Mg^2+^ content in isotonic beverages; Table S3: Experimentally measured pH values of isotonic beverages; Figure S1: Kinetics of standard sodium chloride solutions; Figure S2: Spectrum of light scatter and absorption of isotonic beverages; Figure S3: −log(*I*/*I*_0_) as a function of wavelength for suspensions of erythrocytes in isotonic beverages; Figures S4–S15: Erythrocytes in standard NaCl solutions of different osmolalities.
