# Supplementary material for: Osmolality and Tonicity of Isotonic Beverages
Source: Foods. 2024 May 10;13(10):1483. doi: 10.3390/foods13101483 (PMC11120308; doi:10.3390/foods13101483)
Supplement: Supplementary file 1 [file foods-13-01483-s001.zip › foods-2983103-supplementary.pdf]

Supplementary Information for:

Osmolality and Tonicity of Isotonic Beverages

Table S1. Labelled content of nutrients per 100 mL of isotonic beverage.

Sample	Nutrients	Components	Per 100 mL
sample 1	carbohydrates	fructose	4,2 g
	- of which of which sugars	fructose	4,2 g
	salt		< 0,005 g
	sodium		< 0,002 g
	potassium		
	calcium	calcium lactate	24 mg
	magnesium	magnesium carbonates	11,3 mg
sample 2	carbohydrates	fructose	4 g
	- of which sugars	fructose	4 g
	salt		0 g
	sodium	sodium citrate	/
	potassium	potassium citrate	
	calcium	calcium lactate	
	magnesium	magnesium carbonates	
sample 3	carbohydrates	fructose syrup	5,0 g
	- of which sugars	fructose syrup	2,8 g
	salt		< 0,1 g
	sodium		< 0,04 g
	potassium		
	calcium		
	magnesium	magnesium carbonates	11,3 mg
sample 4	carbohydrates	maltodextrin	5,9 g
	- of which sugars		3,9 g
	salt		0,13 g
	sodium	sodium citrate	0,052 g
	potassium	monopotassium phosphate	
	calcium		
	magnesium	magnesium oxide	

sample 5	carbohydrates	maltodextrin, glucose	4 g
	- of which sugars	glucose	3,5 g
	salt		0,14 g
	sodium	trisodium citrate, sodium benzoate	0,056 g
	potassium	tripotassium citrate, potassium sorbate	68,2 mg
	calcium	calcium lactate	22,6 mg
	magnesium	magnesium carbonate	10,9 mg

sample 6	carbohydrates		4,5 g
	- of which sugars		4,5 g
	salt		< 0,01 g
	sodium		< 0,004 g
	potassium	tripotassium citrate	
	calcium		
	magnesium	magnesium carbonate	11,3 mg

sample 7	carbohydrates	maltodextrin, sucrose, glucose	6,2 g
	- of which sugars	sucrose, glucose	5,4 g
	salt	sodium chloride	0,13 g
	sodium	sodium chloride, sodium citrate, sodium benzoate	50 mg
	potassium	potassium chloride, potassium sorbate	17 mg
	calcium	dicalcium phosphate, calcium lactate	12 mg
	magnesium	magnesium citrate	1,5 mg

sample 8	carbohydrates	fructose	4,0 g
	- of which sugars	fructose	4,0 g
	salt	sodium chloride	0,10 g
	sodium	sodium chloride, trisodium citrate, sodium benzoate,	0,04 g
	potassium	potassium chloride, potassium sorbate	
	calcium	calcium chloride	24 mg
	magnesium	magnesium salt of citric acid	11 mg

sample 9	carbohydrates	glucose, fructose	3,9 g
	- of which sugars	glucose, fructose	3,9 g
	salt	sodium chloride	0,13 g
	sodium	sodium chloride	0,052 g
	potassium		
	calcium		
	magnesium		

sample 10	carbohydrates	maltodextrin, sucrose, glucose syrup	6,9 g
	- of which sugars	sucrose, glucose syrup	5,9 g
	salt	sodium chloride	0,18 g
	sodium	sodium chloride, sodium citrate	0,07 g
	potassium	potassium chloride	
	calcium	phosphorous acid calcium salt	31,0 mg
	magnesium	magnesium carbonate	12,5 mg

sample 11	carbohydrates		5,3 g
	- of which sugars		3,6 g
	salt		contains negligible amounts
	sodium		
	potassium		
	calcium		
	magnesium		

sample 12	carbohydrates		0 g
	- of which sugars		0 g
	salt	sodium chloride	32 mg
	sodium	sodium chloride, sodium citrate, sodium benzoate	12,8 mg
	potassium	potassium sorbate	up to 25 mg
	calcium		
	magnesium	magnesium citrate	12 mg

sample 13	carbohydrates	maltodextrin, sucrose, glucose syrup	7 g
	- of which sugars	sucrose, glucose syrup	5,6 g
	salt	sodium chloride	0,22 g
	sodium	sodium chloride, sodium citrate	0,088 g
	potassium		
	calcium	phosphorous acid calcium salt	32 mg
	magnesium	magnesium carbonate	12,4 mg
sample 14	carbohydrates	maltodextrin	6,5 g
	- of which sugars		5,6 g
	salt		0,03 g
	sodium	sodium citrate	0,012 g
	potassium		
	calcium	calcium lactate	24,0 mg
	magnesium	magnesium carbonate	11,5 mg
sample 15	carbohydrates	maltodextrin, glucose	7,2 g
	- of which sugars	glucose	6,6 g
	salt		0,13 g
	sodium	sodium citrate	0,0504 g
	potassium	potassium chloride	
	calcium	calcium phosphate	
	magnesium	magnesium citrate, magnesium carbonate	30,4 mg
sample 16	carbohydrates		4,4 g
	- of which sugars		4,2 g
	salt		0,043 g
	sodium		0,0172 g
	potassium		
	calcium		
	magnesium		
sample 17	carbohydrates	maltodextrin, glucose	6,7 g
	- of which sugars	glucose	4,4 g
	salt		0,12 g
	sodium	sodium citrate, sodium benzoate	0,048 g
	potassium	potassium citrate, potassium sorbate	
	calcium		
	magnesium		

sample 18	carbohydrates	maltodextrin, glucose	5,1 g
	- of which sugars	glucose	3,8 g
	salt		0,14 g
	sodium	sodium citrate, sodium benzoate	0,056 g
	potassium	potassium sorbate	
	calcium		
	magnesium		

Table S2. Experimentally measured Na^+, K^+, and Mg^{2+} content in isotonic beverages.

Sample	Average Na^+ content (mg/100 mL)	Average K^+ content (mg/100 mL)	Average Mg^{2+} content (mg/100 mL)
1	1 ± 0	0,1 ± 0,0	14,3 ± 1,8
2	12 ± 6	38,3 ± 3,0	9,0 ± 0,9
3	1 ± 0	25,7 ± 1, ±	14,6 ± 1,7
4	53 ± 7	13,7 ± 0,6	5,5 ± 0,4
5	55 ± 3	50,2 ± 12,1	4,8 ± 0,2
6	3 ± 1	78,8 ± 6,2	11,6 ± 0,8
7	61 ± 10	21,3 ± 0,8	1,7 ± 0,0
8	45 ± 8	17,6 ± 1,3	12,5 ± 0,7
9	61 ± 4	1,3 ± 0,2	0,4 ± 0,0
10	94 ± 30	20,9 ± 0,5	13,4 ± 1,1
11	0 ± 0	38,0 ± 1,5	4,2 ± 0,6
12	88 ± 15	8,3 ± 1,1	14,2 ± 0,2
13	98 ± 8	0,2 ± 0,1	14,2 ± 1,9
14	15 ± 2	0,4 ± 0,0	11,9 ± 0,8
15	53 ± 30	24,6 ± 2,8	33,4 ± 4,7
16	10 ± 11	34,3 ± 9,0	4,4 ± 0,5
17	52 ± 23	15,4 ± 1,8	1,7 ± 0,1
18	54 ± 24	7,4 ± 0,9	0,0 ± 0,0

Table S3. Experimentally measured pH values of isotonic beverages.

Sample	pH
1	3,93
2	3,39
3	4,02
4	3,28
5	3,78
6	3,79
7	3,76
8	3,45
9	2,60
10	3,96
11	4,40
12	3,74
13	4,14
14	3,70
15	4,54
16	5,65
17	3,87
18	3,67

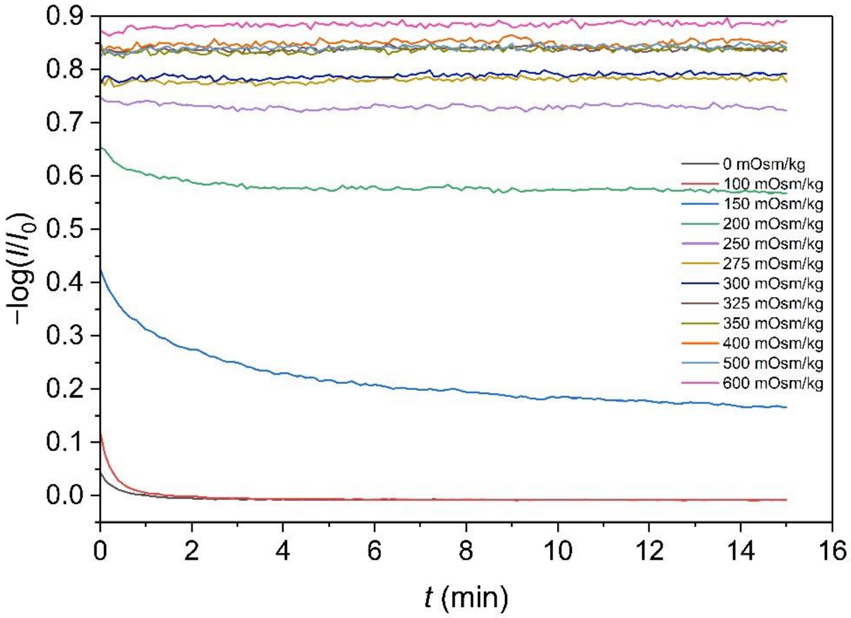

Figure S1. Kinetics of standard sodium chloride solutions. Erythrocytes lyse in 0 mOsm/kg and in 100 mOsm/kg solutions within 1 minute, as the values of $-\log(I/I_0)$ drop to 0. In 150 and 200 mOsm/kg solution, only a part of erythrocytes is lysed, since $-\log(I/I_0)$ does not reach the same low values as in the case of 0 and 100 mOsm/kg solution. $-\log(I/I_0)$ value increases with increasing osmolality, but the differences between the spectra for solutions with osmolality greater than 250 mOsm/kg are negligible and cannot set a clear boundary between isotonic and hypertonic solution.

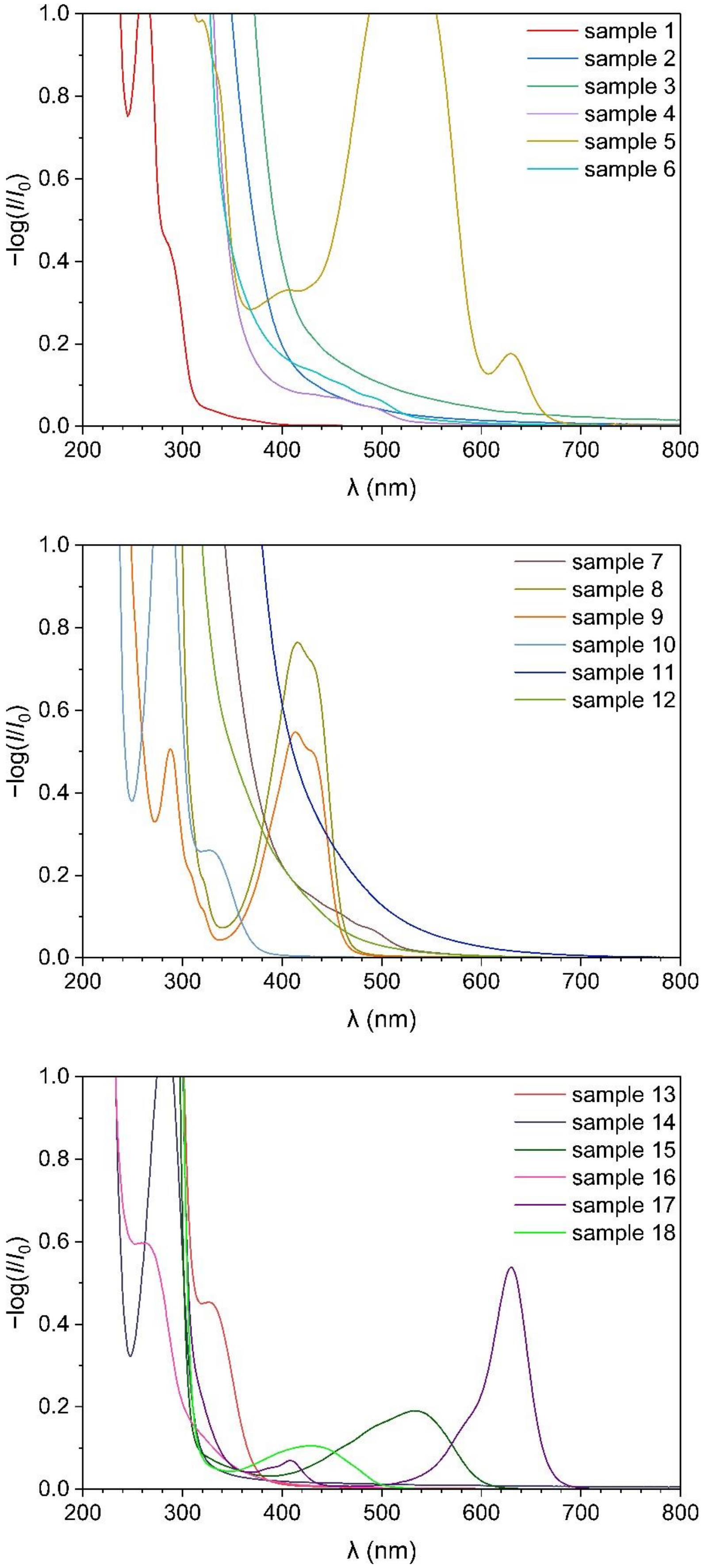

Figure S2. Spectrum of light scatter and absorption of isotonic beverages.

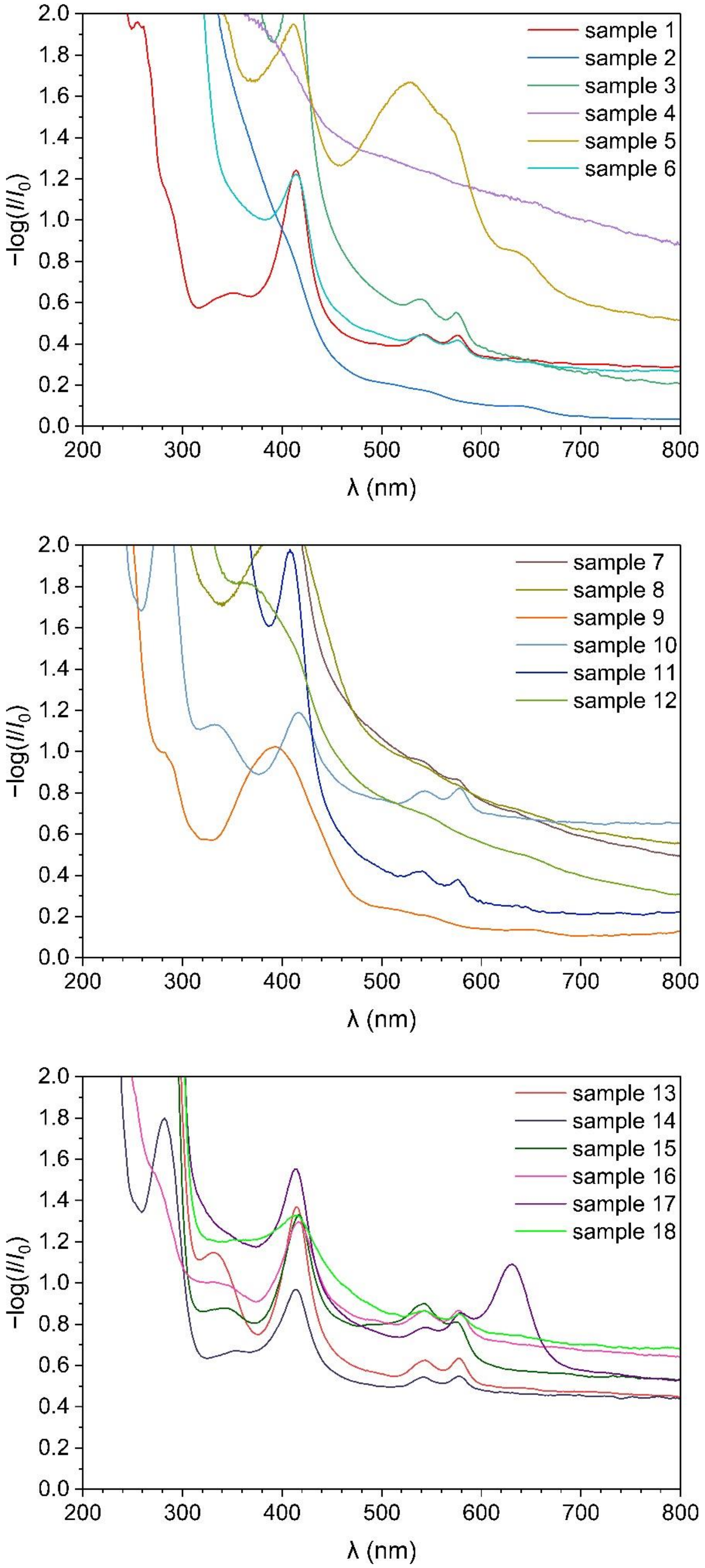

Figure S3. –log(I/I_0) as a function of wavelength for suspensions of erythrocytes in isotonic beverages. Typical peaks for hemoglobin can be observed here, except for samples 2, 4, 7, 8 and 12.

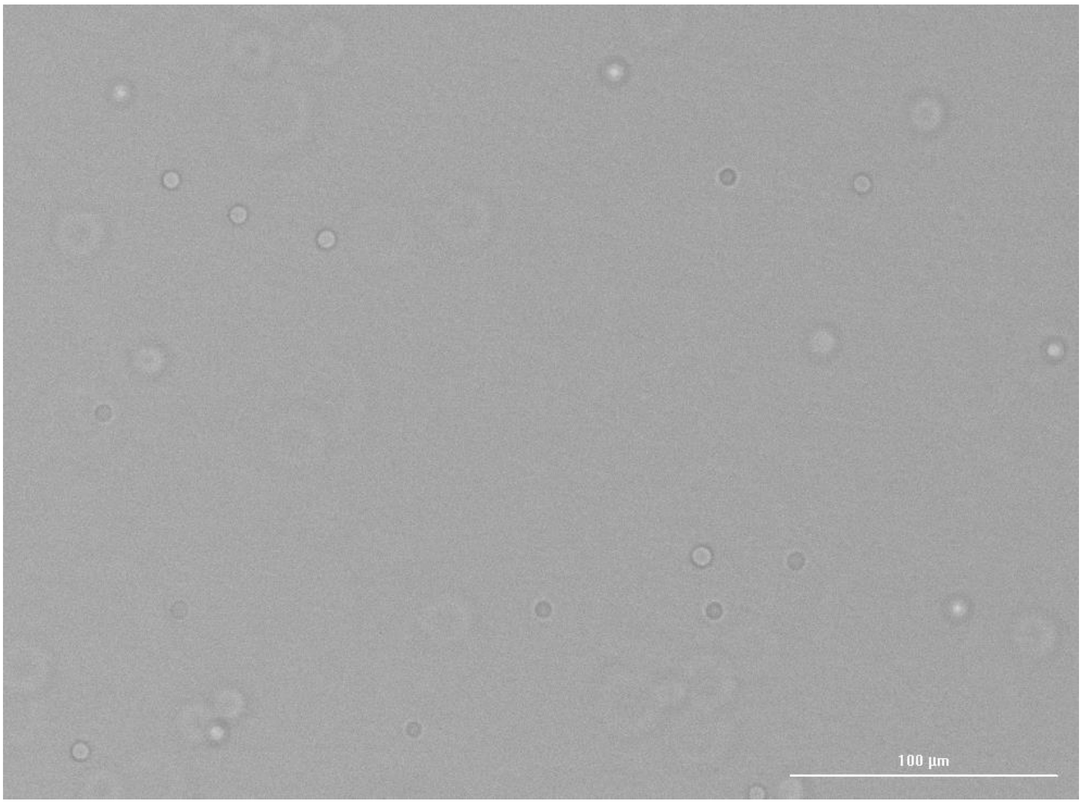

Figure S4. Erythrocytes in 0 mOsm/kg NaCl solution.

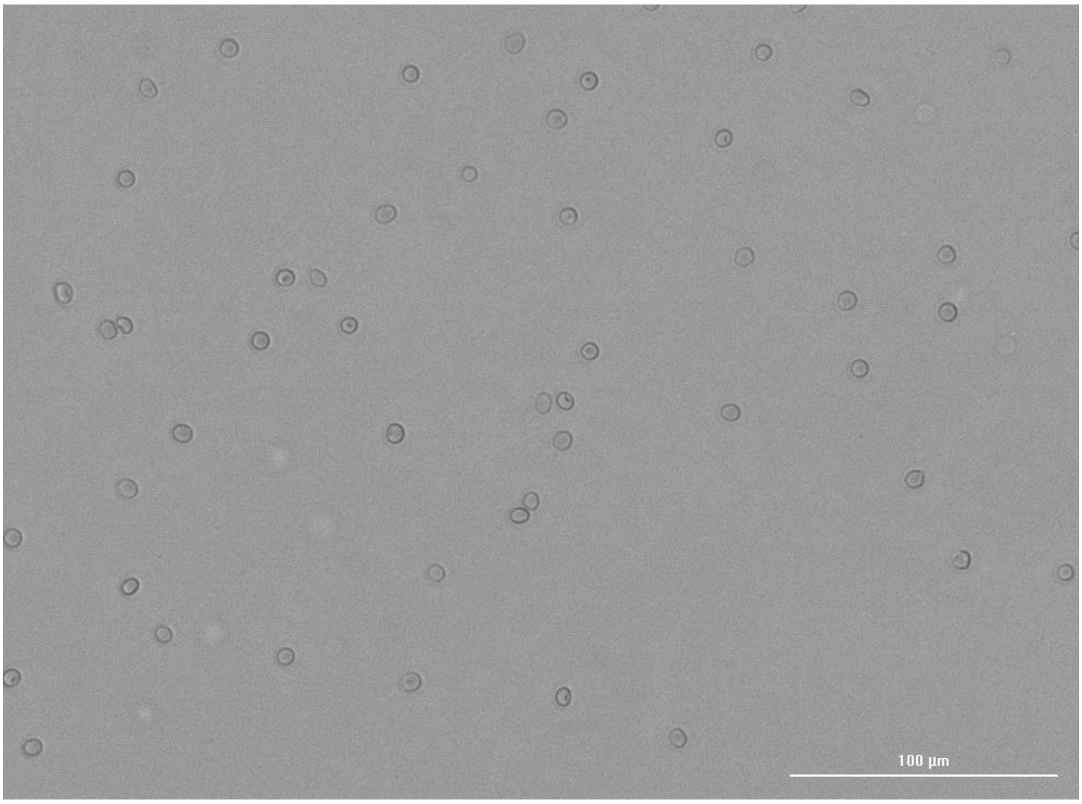

Figure S5. Erythrocytes in 100 mOsm/kg NaCl solution.

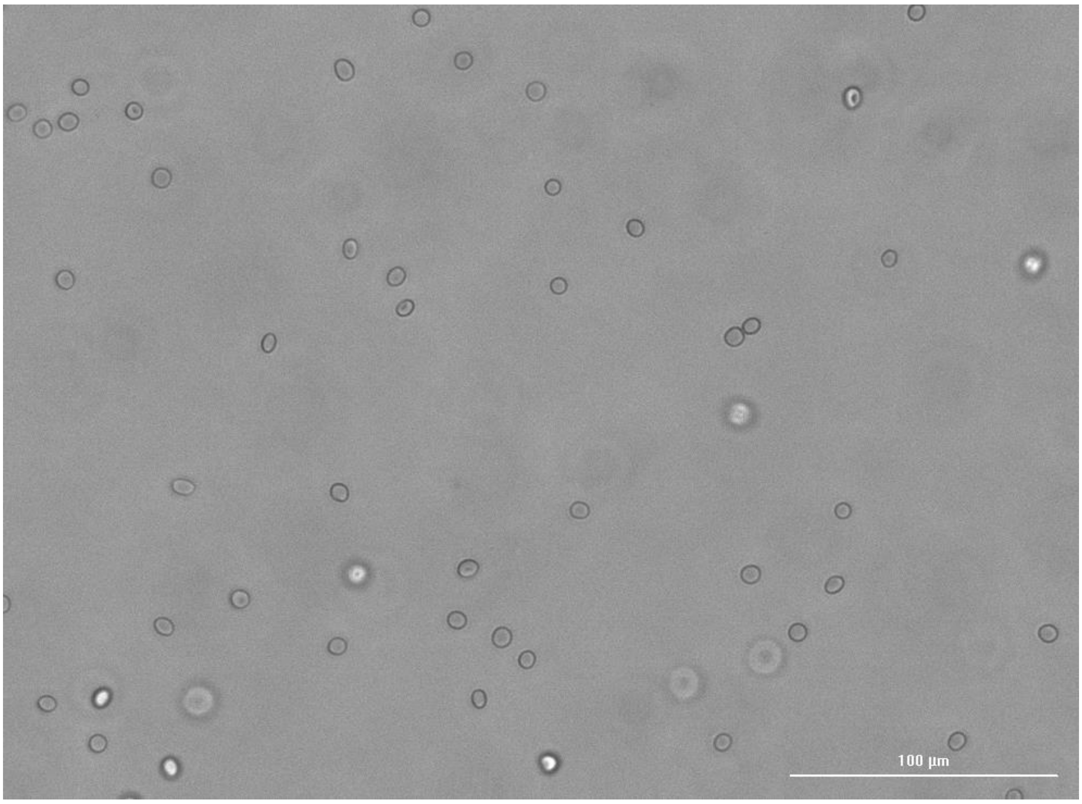

Figure S6. Erythrocytes in 150 mOsm/kg NaCl solution.

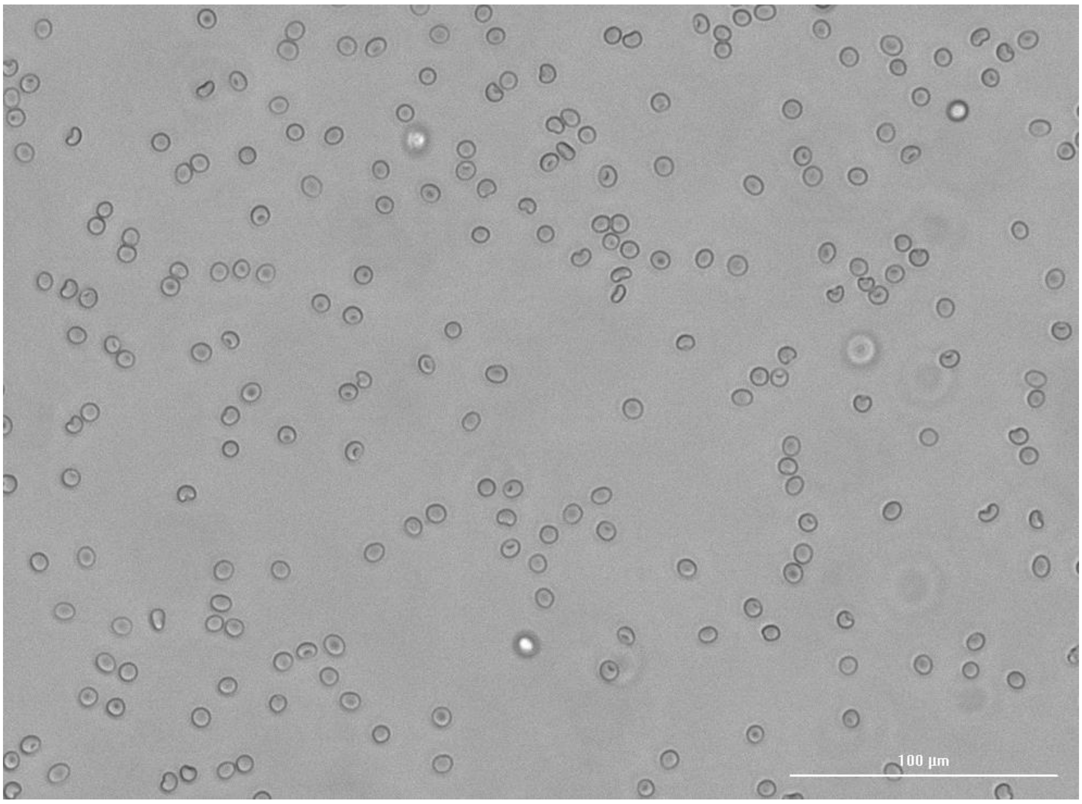

Figure S7. Erythrocytes in 200 mOsm/kg NaCl solution.

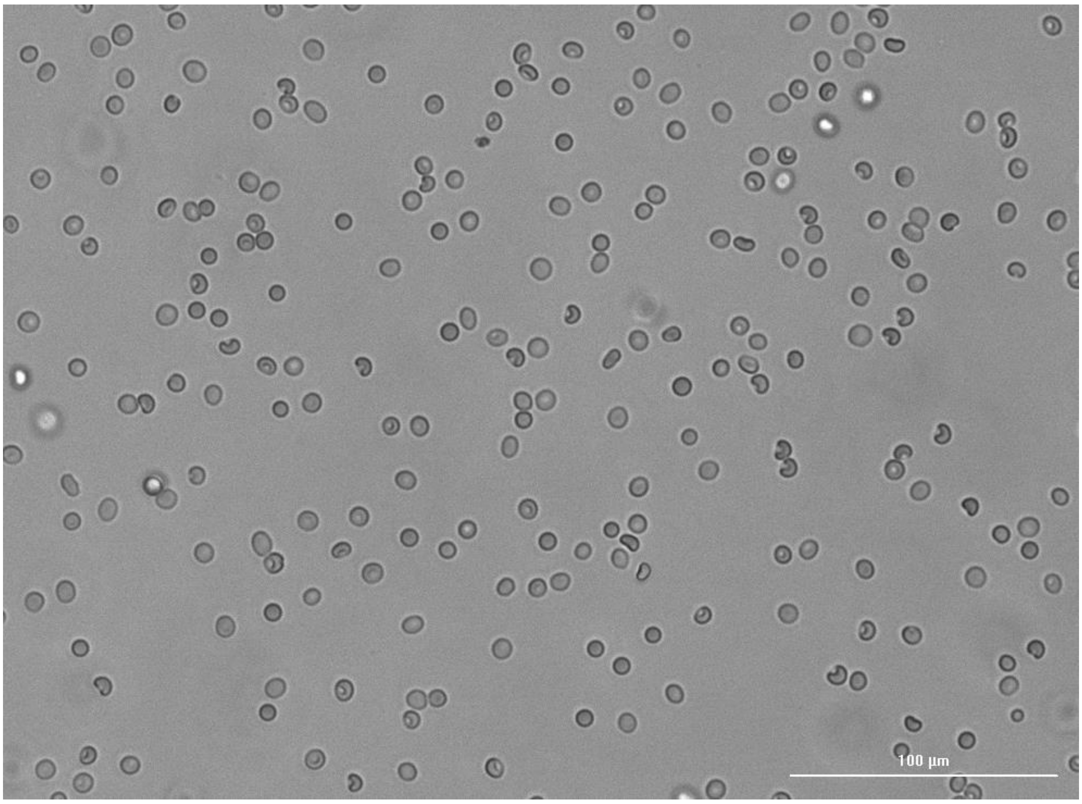

Figure S8. Erythrocytes in 250 mOsm/kg NaCl solution.

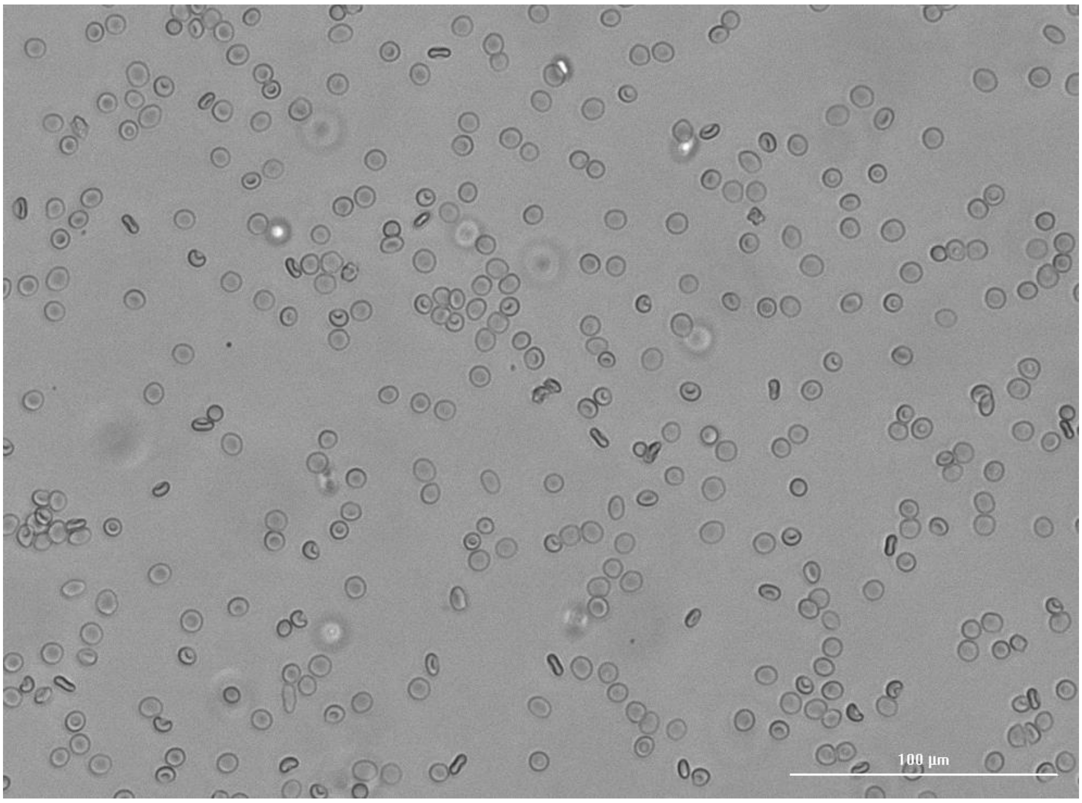

Figure S9. Erythrocytes in 275 mOsm/kg NaCl solution.

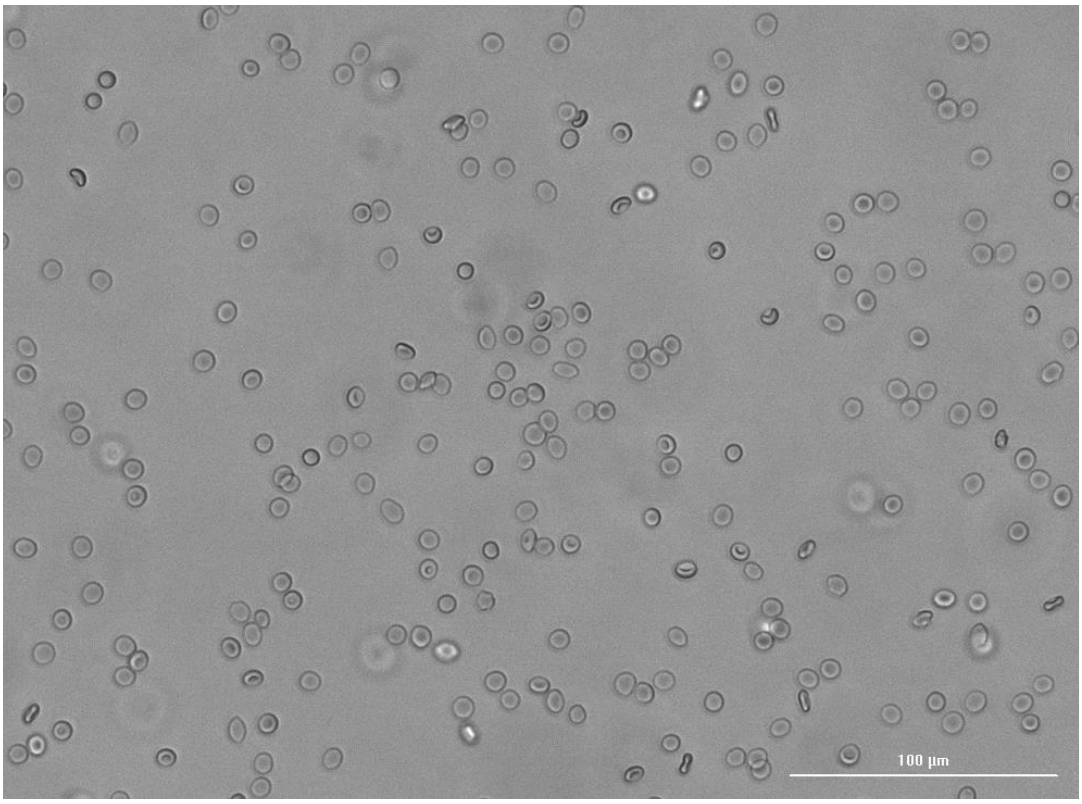

Figure S10. Erythrocytes in 300 mOsm/kg NaCl solution.

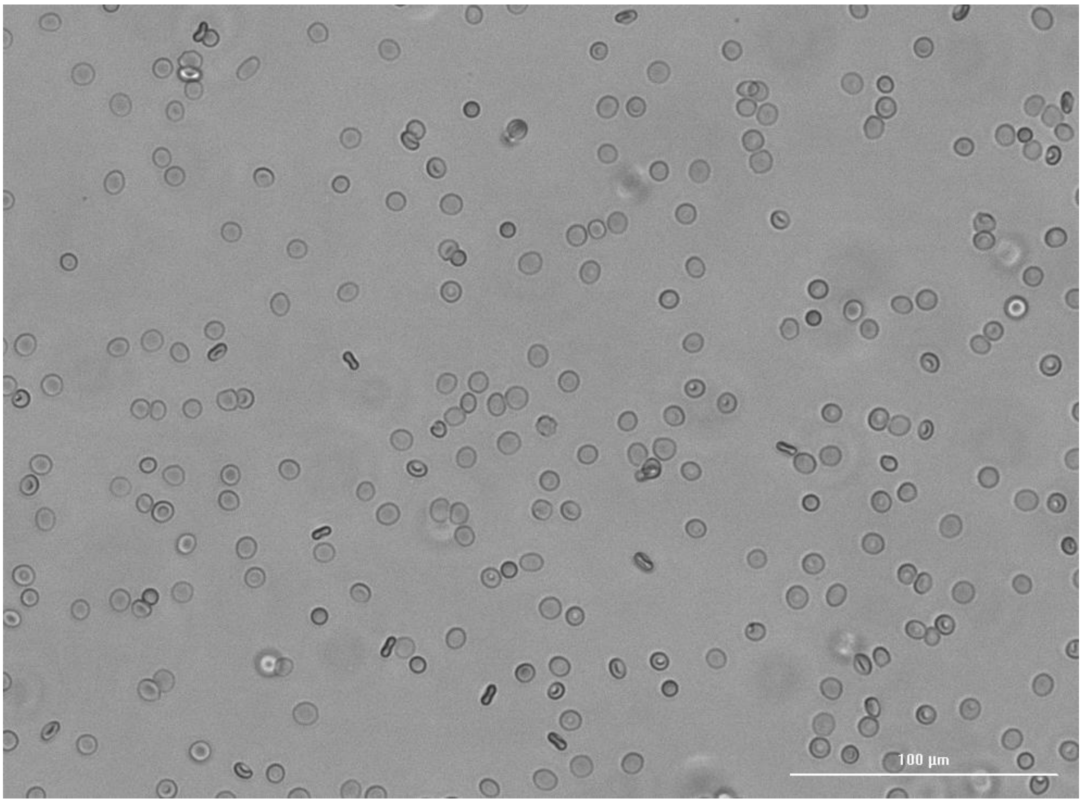

Figure S11. Erythrocytes in 325 mOsm/kg NaCl solution.

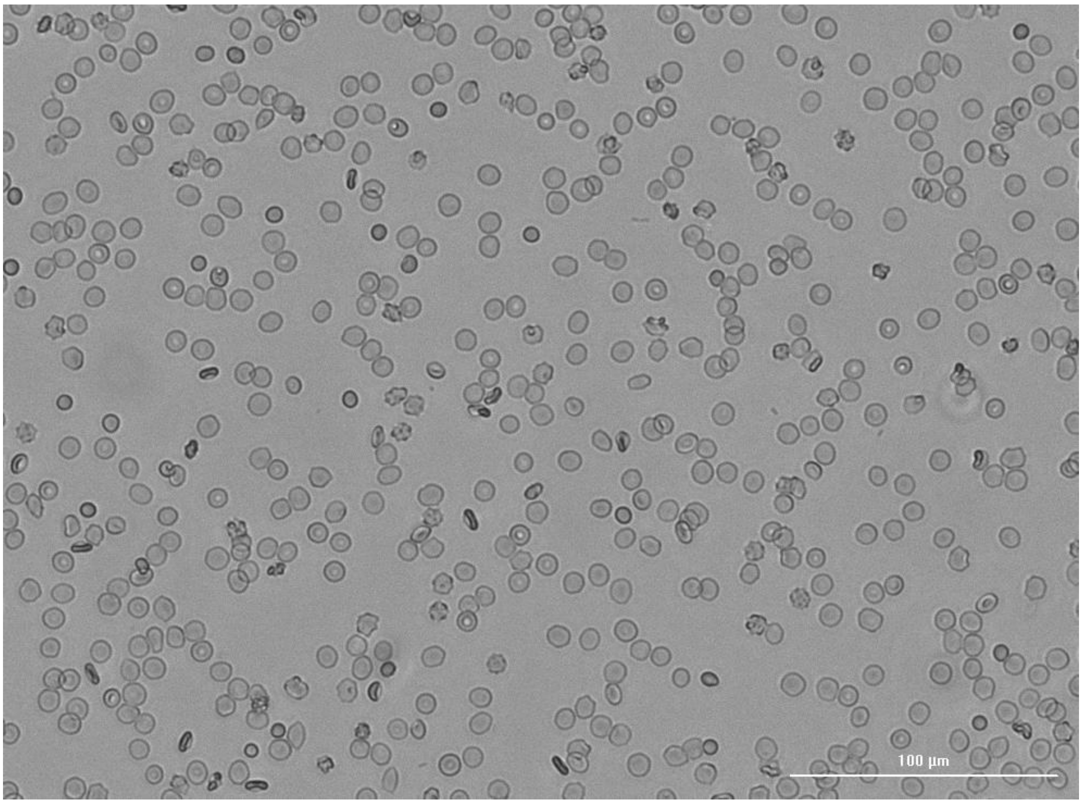

Figure S12. Erythrocytes in 350 mOsm/kg NaCl solution.

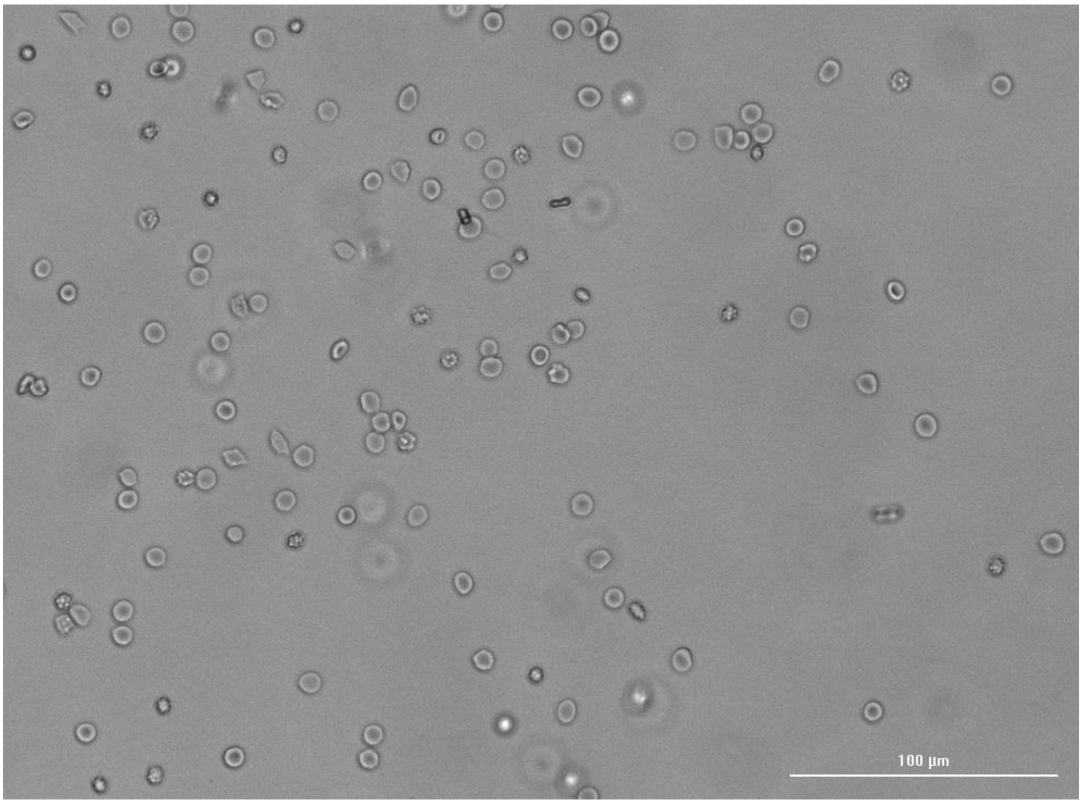

Figure S13. Erythrocytes in 400 mOsm/kg NaCl solution.

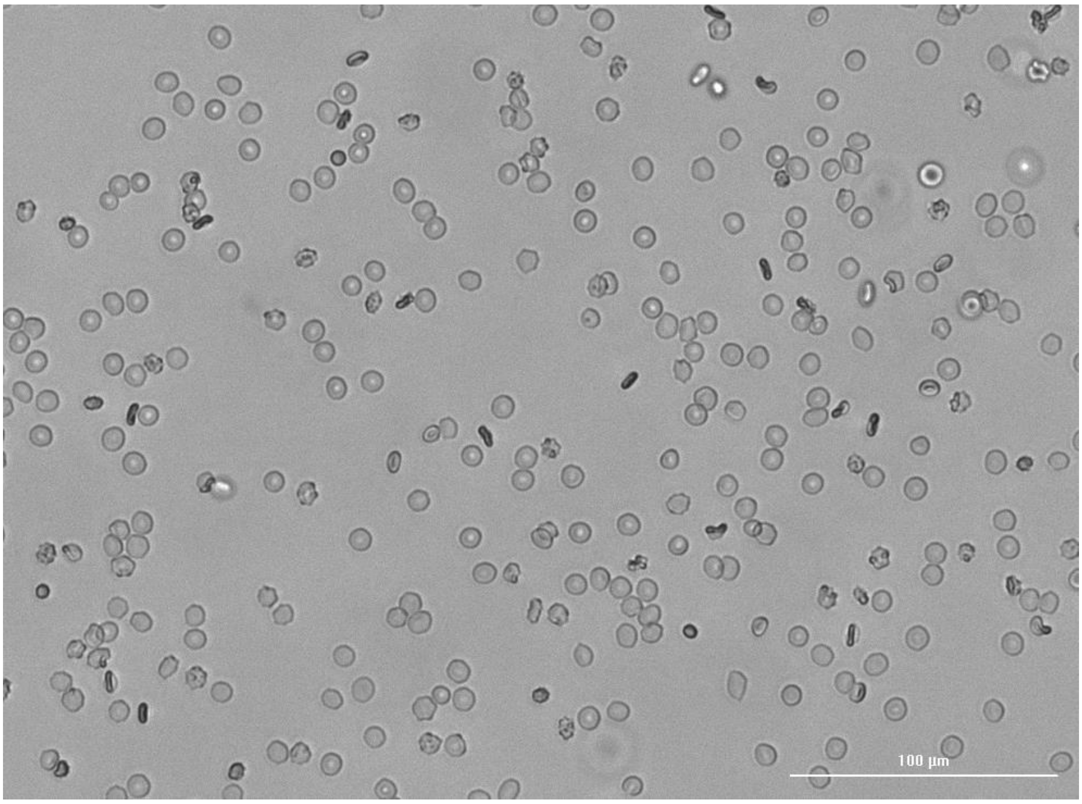

Figure S14. Erythrocytes in 500 mOsm/kg NaCl solution.

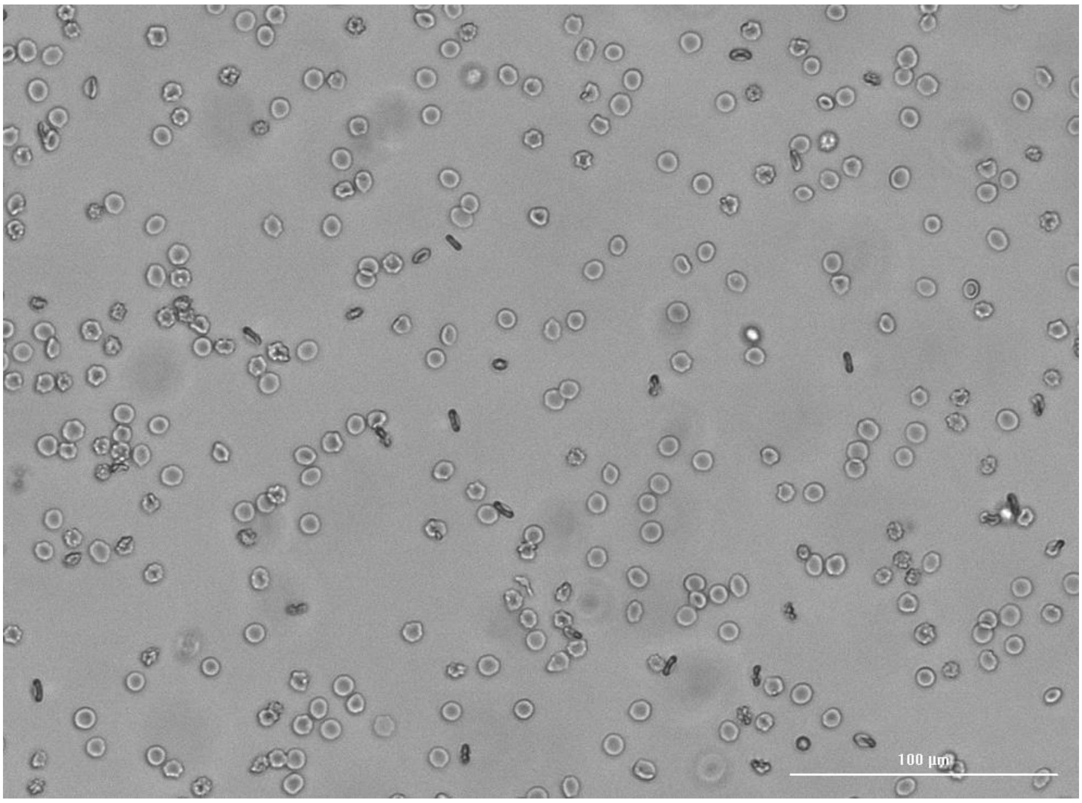

Figure S15. Erythrocytes in 600 mOsm/kg NaCl solution.
